# The African Colonial Enterprise: Experimental Stage

**Published:** 1898-06

**Authors:** 


THE AFRICAN COLONIAL ENTERPRISE: EXPERIMENTAL STAGE. Organizer and General Director, J. Albert Thorne, M. B., C. M., 22 Devonshire Street, Portland Place, London, W., England. Temporary Office of the Committee, 1541 Thompson Street, Philadelphia, Pa., U. S. A.

This pamphlet is an appeal to the friends of the African race in Great Britain and the United States to subscribe funds to bring about a return of the Negro race to its fatherland, Africa.

There are three principal reasons for thus advancing this idea to full accomplishment. First, because the African in civilized countries having the necessary religious knowledge is capable of spreading evangelical knowledge among the savage tribes of Africa, and these tribes are more likely to accept it from those of their own race.

Secondly, because these tribes show a manifest disposition to receive the enlightened men of their own race who may come among them; and,

Thirdly, because their residence among whites has become intolerable by reason of the disabilities, social and political, under which they live, and they are unwilling longer to endure them.

The plan to accomplish their purpose is to acquire 10,000 acres as a basis and to settle upon this tract 100 families carefully selected and well-trained in various branches of industry. The land is to be divided into ten sections of 1,000 acres each, and upon each section ten families are to be planted. Upon each section employment is to be given to at least 100 natives, who are to be properly trained. A fund is to be raised to enable the settlers to reach their destination, and to be maintained for three years. During this period each head of a family is to receive food and clothing for himself and family. At the end of the third year $144 will be given to each head of a family, as it is deemed likely that by that time they will become self-supporting. This arrangement to continue until the seventh year, when the land will be equally divided among the settlers. The place chosen is in British Central Africa, nearer to the east coast than the west, the climate found there being quite healthy. The sum of $120,000 is needed to inaugurate the work. As soon as this sum is collected the author of the pamphlet under review, himself a full-blooded African, will start for the promised land on the "Zambesi" and establish the colony.

It is the firm conviction of the author that "Africa is the only country in the world where his race will ultimately be respected as a race, because it is there only that they can ever hope to hold their own successfully, and with any true dignity against competitive races."

Toward the accomplishment of this purpose, a great one from every point of view, contributions are solicited, which will be carefully administered and fully accounted for by the committee having the matter in charge.

